# Cell senescence contributes to tissue regeneration in zebrafish

**DOI:** 10.1111/acel.13052

**Published:** 2019-10-31

**Authors:** Sabela Da Silva‐Álvarez, Jorge Guerra‐Varela, Daniel Sobrido‐Cameán, Ana Quelle, Antón Barreiro‐Iglesias, Laura Sánchez, Manuel Collado

**Affiliations:** ^1^ Laboratorio de Células Madre en Cáncer y Envejecimiento Instituto de Investigación Sanitaria de Santiago de Compostela (IDIS) Xerencia de Xestión Integrada de Santiago (XXIS/SERGAS) Santiago de Compostela Spain; ^2^ Departamento de Zoología, Genética y Antropología Física Facultad de Veterinaria Universidade de Santiago de Compostela Lugo Spain; ^3^ Geneaqua S.L Lugo Spain; ^4^ Department of Functional Biology Faculty of Biology CIBUS Universidade de Santiago de Compostela Santiago de Compostela Spain

**Keywords:** cellular senescence, regeneration, tissue injury, zebrafish

## Abstract

Cellular senescence is a stress response that limits the proliferation of damaged cells by establishing a permanent cell cycle arrest. Different stimuli can trigger senescence but excessive production or impaired clearance of these cells can lead to their accumulation during aging with deleterious effects. Despite this potential negative side of cell senescence, its physiological role as a pro‐regenerative and morphogenetic force has emerged recently after the identification of programmed cell senescence during embryogenesis and during wound healing and limb regeneration. Here, we explored the conservation of tissue injury‐induced senescence in a model of complex regeneration, the zebrafish. Fin amputation in adult fish led to the appearance of senescent cells at the site of damage, and their removal impaired tissue regeneration. Despite many conceptual similarities, this tissue repair response is different from developmental senescence. Our results lend support to the notion that cell senescence is a positive response promoting tissue repair and homeostasis.

## Graphical abstract

Tissue injury‐induced senescence is conserved in zebrafish. Fin amputation in adult fish lead to the appearance of senescent cells at the site of damage, and their removal impairs tissue regeneration. Tissue injury‐induced senescence is different from developmental senescence.

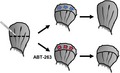

## 1 INTRODUCTION, RESULTS, DISCUSSION

Cellular senescence is a terminal cell response consisting on the implementation of a permanent cell cycle arrest and the acquisition of a secretory phenotype with cell‐to‐cell communication properties (Collado, Blasco, & Serrano, 2007; Muñoz‐Espín & Serrano, 2014). Exhaustion of the proliferative capacity of the cell leads to senescence, and the accumulation of these damaged cells in tissues from old individuals is considered a key element in the process of aging (van Deursen, 2014). Despite this detrimental effect, the senescence response has a beneficial side protecting damaged cells from proliferating. This is considered the basis of its tumor‐suppressive function (Collado et al., 2007; Collado & Serrano, 2010). The recent identification of developmentally programmed cell senescence during embryogenesis expanded our view of the positive activities of this response (Muñoz‐Espín et al., 2013; Storer et al., 2013). Senescence during development promotes cell turnover, tissue remodeling, and, paradoxically, growth. A similar positive pro‐morphogenetic activity for cell senescence has been suggested to operate during skin wound healing in mice (Demaria et al., 2014) and during limb regeneration in salamanders (Yun, Davaapil, & Brockes, 2015). Senescent cells seem to appear at wound sites after injury to help promote optimal wound healing (Yun, 2018).

**Figure 1:**
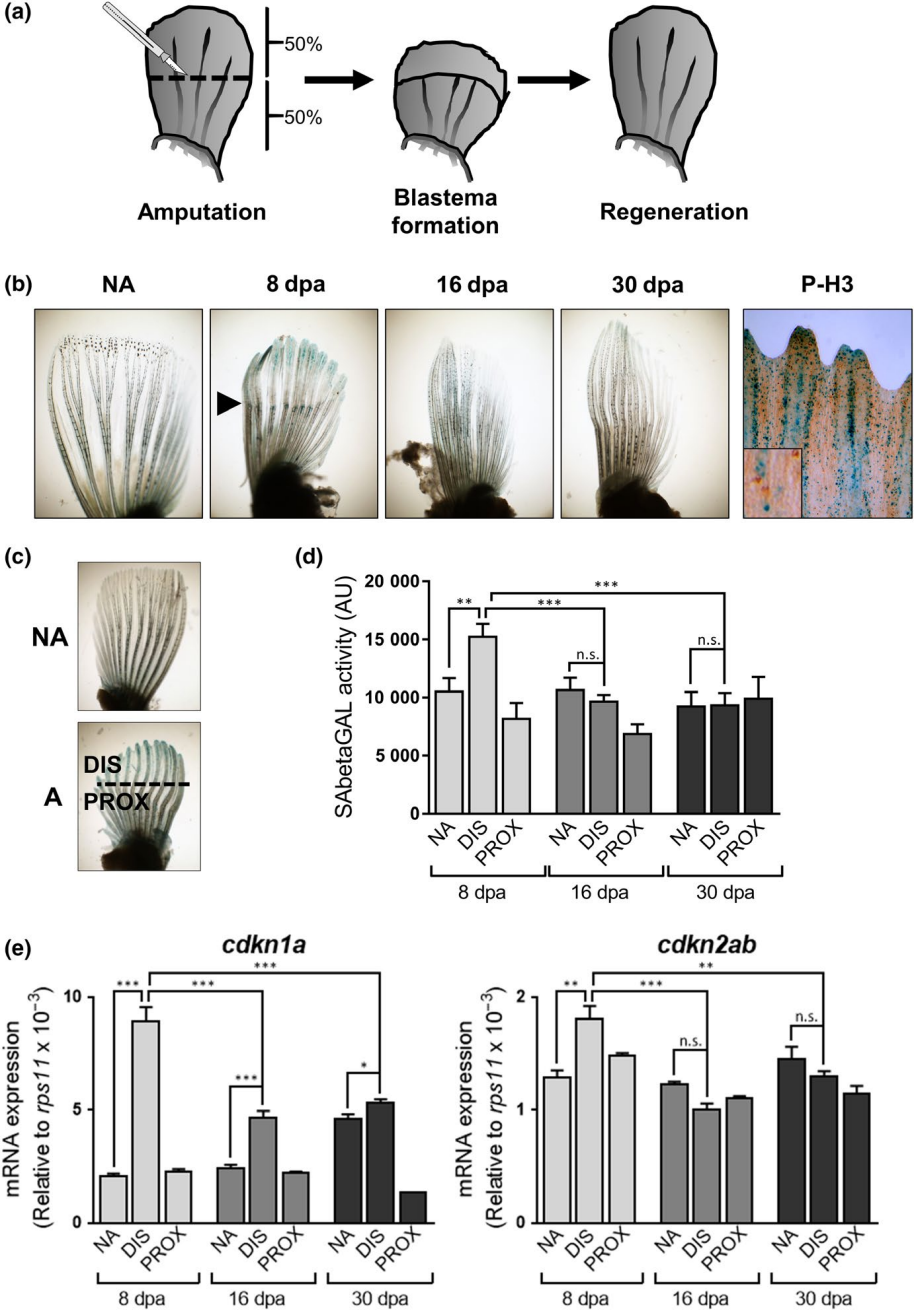
Pectoral fin amputation induces features of cell senescence. (a) Schematic representation of the fin amputation system used throughout the study. (b) Representative photomicrographs of fins stained for SAbetaGal or phospho‐histone 3 (P‐H3, right panel) after amputations (NA: nonamputated; 8, 16, and 30 dpa: days postamputation). Co‐staining of P‐H3 was done at 8 dpa. Arrowhead shows the amputation plane. (c) Schematic representation showing the different types of samples used in the study (NA: nonamputated; A: amputated; DIS: distal area; PROX: proximal). (d) SAbetaGal activity measured using Galacton substrate after 8, 16, and 30 days postamputation (dpa) (from 5–10 animals per condition). (e) Expression levels by QPCR of *cdkn1a* (left panel) and *cdkn2ab* (right panel) genes relative to the housekeeping gene *rps11* after 8, 16, and 30 days postamputation (dpa). Results are presented as mean ± *SD* ***p < .001, **p < .01,*p < .05, n.s. nonsignificant

Here, we decided to evaluate the senescence response in the context of tissue injury using an animal model of complex tissue regeneration, the zebrafish. To study senescence after tissue damage, we amputated the pectoral fin of adult fish (around 1 year old) at approximately 50% of its length and followed regeneration with time (Figure 1a). We stained fins for senescence‐associated beta‐galactosidase (SAbetaGal), the most widely used marker of senescence (Dimri et al., 1995), after 8, 16, or 30 days postamputation (dpa), a time point in which fins were completely regenerated. Control stainings were performed on the contralateral unamputated fin or immediately after amputation to discard artifacts derived from unspecific staining of damaged tissue. Fins at 8 dpa showed intense blue staining compared with light blue at 16 dpa and completely absent staining at 30 dpa (Figure 1b). Immunohistochemical co‐staining with phospho‐histone 3 (P‐H3), a marker of proliferation, at 8 dpa confirmed that the SAbetaGal‐positive cells were not proliferating (Figure 1b). To further confirm these results, we used an alternative senescence detection method more amenable for quantification, utilizing Galacton, a chemiluminescent substrate (Bassaneze, Miyakawa, & Krieger, 2008). We collected amputated and nonamputated fins at different times during regeneration and split the amputated fins into proximal (closer to the body) and distal (the regenerated area) parts (Figure 1c). Again, we observed that 8 dpa was the time point that produced a stronger SAbetaGal reaction and this activity was restricted to the distal part of the fin, the area where regeneration takes place (Figure 1d). In contrast, the proximal area of the 8 dpa fin and the distal or proximal areas of 16 and 30 dpa fins were mostly negative (Figure 1d).

We also extracted RNA from amputated distal and proximal fins and unamputated fins, to check for the expression of some genes that have been linked to the induction of senescence in different species (Collado & Serrano, 2006; Hernandez‐Segura, Nehme, & Demaria, 2018) and in zebrafish (Donnini et al., 2010; Xia et al., 2014). Similar to our results with the SAbetaGal detection, the distal part of 8 dpa fins showed higher expression levels of *cdkn1a* and *cdkn2ab* than the proximal part of amputated fins or the unamputated contralateral fin (Figure 1e). The expression of these senescence markers returned to normal levels after 16 and 30 dpa, in line with our observations using SAbetaGal.

In summary, these results support the notion of a transient induction of cell senescence during fin regeneration as judged by increased SAbetaGal activity and upregulation of the expression of key senescence genes such as *cdkn1a* and *cdkn2ab*. A similar transient induction of senescence has been previously reported during zebrafish heart injury and regeneration (Bednarek et al., 2015).

**Figure 2:**
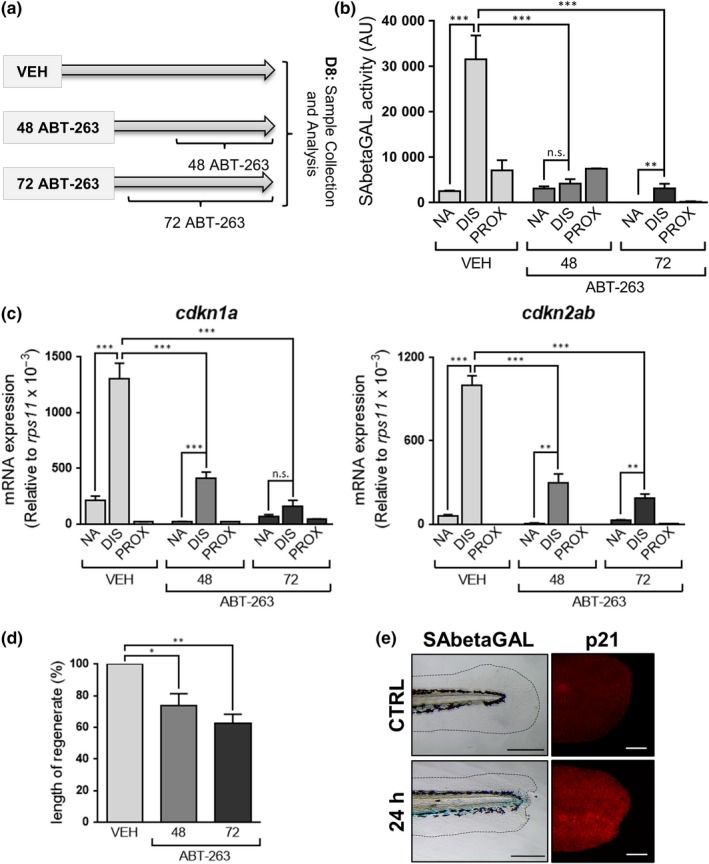
Removal of senescent cells impairs fin regeneration. (a) Schematic representation of the experimental strategy followed to analyze the effect of removing senescent cells from amputated fins after incubation with ABT‐263 for 48 or 72 hr, or treated with vehicle (VEH). (b) SAbetaGal activity measured using Galacton substrate at 8 days postamputation and after treatment with ABT‐263 for 48 or 72 hr, or with vehicle (VEH) (from 5–10 animals per condition). (c) Expression levels by QPCR of *cdkn1a* (left panel) and *cdkn2ab* (right panel) genes relative to the housekeeping gene *rps11* at 8 dpa and after treatment with ABT‐263 for 48 or 72 hr, or with vehicle (VEH). (d) Length of regenerate (%) reached by amputated fins at 8 days postamputation and after treatment with ABT‐263 relative to untreated amputated fins (five animals per group). (e) Representative photomicrographs of larval fins stained for SAbetaGal or p21, 24 hr after amputation and control fin (CTRL). Scale bars: SAbetaGAL: 200 µm; p21: 75 µm. Results are presented as mean ± *SD* ***p < .001, **p < .01, *p < .05, n.s. nonsignificant

Triggering senescence after tissue injury could have positive or negative effects on the regenerative capacity of the damaged tissue, due to its potential pro‐regenerative and anti‐proliferative activities, respectively (He & Sharpless, 2017). To directly assess the role of senescence induction during fin amputation, we decided to induce the removal of these senescent cells from amputated fins. For this, we treated fish for 48 or 72 hr with ABT‐263 (Navitoclax), a senolytic compound that by inhibiting the Bcl‐2 antiapoptotic family of proteins triggers specifically the death of the senescent cells (Chang et al., 2016). We determined the activity of the SAbetaGal enzyme in extracts from unamputated fins as control and from the proximal and distal regions of amputated fins that were previously treated with ABT‐263 for 48 or 72 hr or that were incubated with vehicle as a negative control (Figure 2a). ABT‐263 treatment caused a reduction in SAbetaGal staining and a concomitant induction of apoptosis in the regenerating area, as determined by TUNEL staining (Figure S1A–C). We quantified SAbetaGal activity at 8 dpa, the day at which we had observed the peak of senescence induction. We confirmed the induction of SAbetaGal activity in the vehicle‐treated fish and observed that the activity present in the extracts from the regenerating (distal) region was blunted by the ABT‐263 treatment (Figure 2b). Furthermore, mRNA expression analysis of *cdkn1a* and *cdkn2ab* after ABT‐263 treatment also confirmed the drastic reduction in the levels of these senescence markers at the regenerating area after 48 and 72 hr of incubation (Figure 2c).

These results clearly show that it is possible to remove senescent cells from the regenerating area of injured fins by treating fish with the senolytic compound ABT‐263, so we wondered what was the effect on regeneration. For this, we determined the regenerative capacity by measuring the length of regenerate at 8 dpa in fish treated with ABT‐263 for 48 or 72 hr or vehicle. This analysis revealed that the removal of senescent cells by ABT‐263 treatment clearly impaired regeneration, with amputated fins in fish treated with ABT‐263 showing a clear reduction in the length of regenerate compared with the one reached in vehicle‐treated animals (73.67% ± 14.92% and 62.57% ± 12.87% after 48 or 72 hr, respectively) (Figure S1D and Figure 2d). Apoptosis has been shown to be a crucial process during fin regeneration in zebrafish (Vriz, Reiter, & Galliot, 2014). Since ABT‐263 inhibits proteins of the Bcl‐2 family and this could interfere with the pro‐regenerative apoptosis response, we decided to use an alternative senolytic treatment, quercetin (Zhu et al., 2015). Treatment with quercetin led to a similar reduction of SAbetaGal staining and an impaired regeneration (Figure S1E–G).

The recent discovery of cell senescence during embryo development as part of a developmental program points to a role for senescence as a morphogenetic and proliferative force (Yun, 2018). Senescence induction during adult tissue injury could have resulted from the evolutionary co‐option of this developmental program retained during adulthood. To further clarify the occurrence of senescence during development and tissue injury, we tested senescence induction in 3 dpf fish larvae after a complete spinal cord transection at the level of the anal pore which also damaged the surrounding body wall (muscles and skin). At 2 days postlesion (5 dpf animals), a very strong SAbetaGal staining appeared in the skin and body‐wall muscles only at the injury site in lesioned animals and not in control unlesioned animals, or in portions of the trunk away from the injury site in lesioned animals (Figure S2H). Thus, tissue injury‐induced senescence is not an exclusive property of fin amputation, since a different kind of traumatic injury induces also cellular senescence in the skin and muscles of the trunk in zebrafish.

Interestingly, and in contrast to limbs in mice (Muñoz‐Espín et al., 2013; Storer et al., 2013), fins are negative for senescence markers during zebrafish development (Villiard et al., 2017). However, amputation of the caudal fin of 2 dpf larvae produced a clearly positive reaction for SAbetaGal activity and p21 expression (the product of *cdkn1a* gene) (Figure 2e, and Figure S1I). These results suggest that developmental senescence and tissue regenerative cell senescence are different cell responses triggered by different stimuli that might share some features such as their positive role promoting tissue remodeling and growth. However, our data do not allow us to distinguish between a role in wound healing or during regeneration.

In summary, our results lend support to the notion that tissue injury‐induced senescence is a positive response that promotes regeneration not only during mouse skin wound healing or salamander limb amputation, but also in zebrafish, a widely used animal model of complex regeneration.

## CONFLICT OF INTEREST

Authors declare no conflict of interest.

## AUTHOR CONTRIBUTIONS

S.DS.‐A. performed and interpreted most of the experiments and helped writing the manuscript. J.G.‐V., D.S.‐C. and A.Q. helped with experiments. A.B.‐I., L.S. and M.C. designed the experiments, interpreted the results and wrote the manuscript.
