# Supplementary material for: Cell senescence contributes to tissue regeneration in zebrafish
Source: Aging Cell. 2019 Oct 31;19(1):e13052. doi: 10.1111/acel.13052 (PMC6974711; doi:10.1111/acel.13052)
Supplement: Supplementary file 1 [file ACEL-19-e13052-s001.docx]

**SUPPLEMENTAL INFORMATION**

**MATERIALS AND METHODS**

**Animals**

Wild-type female adult (12 months) zebrafish were from the AB line. Embryos were obtained from breeding of wild-type adult zebrafish. Animals were maintained in a controlled environment in a 14h light/10h dark cycle at 28°C (Westerfield et al, 2007). The protocols used in this study were performed in compliance with the EU animal experimentation regulation (EU, 2010) and were approved by the Bioethics Committee for Animal Experimentation CEEA-LU (Universidade de Santiago de Compostela, Spain).

**Pectoral fin amputation**

To perform pectoral fin amputation, animals were anesthetized with Tricaine and 50% of the fin length was amputated using surgical scissors. We used groups of 18 animals per condition. Samples were collected after regeneration using a scalpel.

**Spinal cord injury**

Complete spinal cord transections were performed at the level of the anal pore in 3 days post-fertilization (dpf) animals using sharp 30-gauge injection needles as previously described (Ohnmacht et al., 2016). This traumatic injury causes a complete transection of the spinal cord and of the surrounding body wall muscles and skin leaving the notochord and main blood vessels intact. 2 days after the injury the larvae were processed for SAbetaGal and cryostat sectioning.

**Senescence-associated β-galactosidase activity**

Determination of SAbetaGal activity was performed through the widely used X-gal staining method and by a chemiluminescent reaction employing Galacton substrate. For the chromogenic reaction, we performed whole-mount stainings essentially as originally described (Dimri et al., 1995). Briefly, adult pectoral fins or larvae were fixed for 20 min (room temperature) in 2% formaldehyde/0.2% glutaraldehyde, washed and incubated overnight at 37ºC with freshly made SAbetaGal solution: 1 mg of 5-bromo-4-chloro-3-indolyl beta-D-galactoside (X-Gal) per mL (Fisher Scientific), 40 mM citric acid/sodium phosphate pH 6.0, 5 mM K_3_Fe[CN]_6_ , 5 mM K_4_Fe[CN]_6_, 150 mM NaCl, and 2 mM MgCl_2_. After staining, adult pectoral fins or larvae were washed with PBS and photographed under an inverted microscope. Quantification was performed using the FIJI image processing software based on ImageJ, by measuring the staining within the regenerated area.

For chemiluminescent detection, 5μl of protein extracts from adult pectoral fins (from 5-10 animals per condition) were used to perform this assay with the Galacto-Light Plus™ beta-Galactosidase Reporter Gene Assay System Kit (Applied Biosystems) according to the manufacturer’s instructions but using a citric acid/sodium phosphate buffer at pH 6.0. Briefly, the tissue was disrupted in Lysis Solution using TissueLyser II. To prepare the reaction buffer, the substrate was diluted 1:100 in 40 mM citric acid/sodium phosphate buffer, pH 6.0. Extracts, 5 μL, were incubated with 70 μL of Reaction Buffer in a white 96w plate and left for 2 hours at room temperature. After incubation, 100 μl of Accelerator-II was added and the signal was measured for 1 second per well using the Mithras LB 940 multimode microplate reader (Berthold Technologies). Luminescence values were referred to the protein concentration, as determined using Bradford Protein Assay Kit (Bio-Rad).

**Cryostat sectioning**

After SAbetaGal staining, larvae with a spinal cord injury were rinsed in PBS, cryoprotected with 30% sucrose in PBS overnight at 4 ºC, embedded in Tissue Tek (Sakura, Torrance, CA, USA), frozen in liquid nitrogen-cooled isopentane and cut serially on a cryostat (16 μm sections) in the transverse plane. Sections were mounted on Superfrost® Plus glass slides (Menzel, Braunschweig, Germany) using Mowiol® (Sigma). Photomicrographs were taken with an Olympus microscope.

**p21 immunofluorescence in larvae**

For immunofluorescence, control and amputated larvae were fixed by immersion in 4% paraformaldehyde (PFA) in PBS for 4 hours at 4°C. The samples were then rinsed in PBS, incubated in a collagenase solution (2 mg/mL in PBS) for 10 minutes, in a glycine solution (50 mM in PBS) for 10 minutes and incubated with a mouse monoclonal anti-p21 (CDKN1A; clone F-5; dilution 1:500; Santa Cruz, Dallas, Texas; Cat#: sc-6246; RRID: AB_628073; immunogen: mouse p21) at 4°C overnight. The primary antibody was diluted in PBS containing 15% normal goat serum and 0.2% Triton X-100 as detergent. Larvae were rinsed in PBS and incubated overnight at 4°C with a Cy3-conjugated goat anti-mouse antibody (1:200; Millipore; Burlington, MA). Larvae were then rinsed in PBS and mounted with 70% glycerol in PBS. Photomicrographs were taken with an SP2 Leica confocal microscope.

**P-H3 immunohistochemistry in adult pectoral fins**

After SAbetaGal staining, some amputated pectoral fins of adult animals were rinsed in PBS and pretreated with 10% H_2_O_2_ in PBS (to block endogenous peroxidase) before being incubated with a rabbit polyclonal anti-P-H3 antibody (dilution 1:100; Santa Cruz, Dallas, Texas; Cat#: sc-8656-R; RRID: AB_653256; immunogen: phospho(Ser10)-histone 3) at 4°C overnight. The primary antibody was diluted in PBS containing 15% normal goat serum and 0.2% Triton X-100 as detergent. Then, the fins were rinsed in PBS and incubated overnight at 4°C at room temperature with an HRP-conjugated goat anti-rabbit antibody (1:200; Bio-Rad; Hercules, CA) and the staining was developed using 0.6 mg/ml 3-3′-diaminobenzidine (DAB, Sigma) and 0.003% H_2_O_2_. The staining was stopped with PBS and the fins were mounted with 70% glycerol in PBS. Photomicrographs were taken with an SP2 Leica confocal microscope.

**ABT-263 and Quercetin treatments**

ABT-263 (Navitoclax; a generous gift from Abbvie) and Quercetin (a kind gift from Dr Mabel Loza, CIMUS, USC) were directly added to the water to a final concentration of 2 μg/mL or 10 mg/mL, respectively, every 24h for 48 or 72h for ABT-263 or only for 72h for Quercetin. After treatment, larvae were fixed and stained for SAbetaGal (20 animals per condition) or TUNEL labelling, or were used to extract RNA for qPCR.

**TUNEL assay**

In order to detect DNA strand breaks as a sign of cell death, terminal deoxynucleotidyl transferase-mediated dUTP nick end labelling (TUNEL) staining was performed in pectoral fins of control (n = 4) and ABT-263 treated (n = 4) animals. The pectoral fins were fixed by immersion in 4% paraformaldehyde in PBS for 4 hours. After fixation, the tissue was washed in PBS. The TUNEL staining was performed according to the manufacturer´s protocol with minor modifications (*In situ* Cell Death Detection Kit, TMR red; Roche, Mannheim, Germany). Briefly, the fins were incubated in a Proteinase K (Sigma) solution (2.5 µL of 20 mg/mL Proteinase K in 10 mL of PBS) for 10 minutes at 37ºC followed by brief washes in PBS and further fixation in 4% paraformaldehyde in PBS for 15 minutes. After several washes in PBS, the pectoral fins were incubated in the TUNEL reaction mix, containing the Label Solution (TMR red labelled nucleotides) and Enzyme Solution (Terminal deoxynucleotidyl transferase), overnight at RT. Fins were washed in PBS and mounted with 70% glycerol in PBS. Photomicrographs were taken with an SP2 Leica confocal microscope. The total number of TUNEL positive nuclei in the distal 150 µm of each pectoral fin was manually counted using Image J.

**Quantitative RT-PCR**

To measure RNA expression, samples (pools from 10 adult animals) were disrupted using TissueLyser II and total RNA was extracted using the NucleoSpin® RNA kit (Macherey-Nagel) following the indications of the provider and DNAse treatment. After RNA quantification on nanodrop, the RNA was retrotranscribed into cDNA according to the manufacturer’s protocol (High-Capacity cDNA Reverse Transcription Kit, Applied Biosystems). Quantitative Real Time-PCR was performed using SYBR Green Power PCR Master Mix (Applied Biosystems) in an AriaMx real-time PCR system (Agilent technologies). Relative quantitative RNA was normalized using the housekeeping gene *rps11.* The primers used for Quantitative Real Time-PCR were:

*rps11*-F: 5’-ACAGAAATGCCCCTTCACTG-3’

*rps11*-R: 5’-GCCTCTTCTCAAAACGGTTG-3’

*cdkn1a*-F: 5’-CGCAAACAGACCAACATCAC-3’

*cdkn1a*-R: 5’-ATGCAGCTCCAGACAGATGA-3’

*cdkn2ab*-F: 5’-CCGCACGGTGTCAATGAATC-3’

*cdkn2ab*-R: 5’-ATTTTCCCCCTCTCCAGGTG-3’

**Length of regenerate**

Quantification of the length of regenerate at 8dpa after treatment or not with ABT-263 or Quercetin were performed by measuring the distance from the amputation plane to the distal tips of ten fin rays from each animal using ImageJ (5 animals per group).

**Statistical analyses**

The statistical significance of the data obtained was analyzed using the two-tailed *Student's* *t*-test, Mann Whitney U test (for non-normally distributed data) or the *Fisher's* exact test: *** *p* < 0.001; ** *p* < 0.01; * *p* < 0.05; n.s. not significant.

**REFERENCES FOR METHODS**

Dimri, G.P., Lee, X., Basile, G., Acosta, M., Scott, G., Roskelley, C., Medrano, E.E., Linskens, M., Rubelj, I., and Pereira-Smith, O. (1995). A biomarker that identifies senescent human cells in culture and in aging skin in vivo. Proc. Natl. Acad. Sci. U. S. A. *92*, 9363–9367.

Ohnmacht, J., Yang, Y., Maurer, G.W., Barreiro-Iglesias, A., Tsarouchas, T.M., Wehner, D., Sieger, D., Becker, C.G., and Becker, T. (2016). Spinal motor neurons are regenerated after mechanical lesion and genetic ablation in larval zebrafish. Development *143*, 1464–1474.

Westerfield M: The Zebrafish Book. A Guide for the Laboratory Use of Zebrafish (Danio rerio), 5th Edition. Univ Oregon Press Eugene, OR, 2007


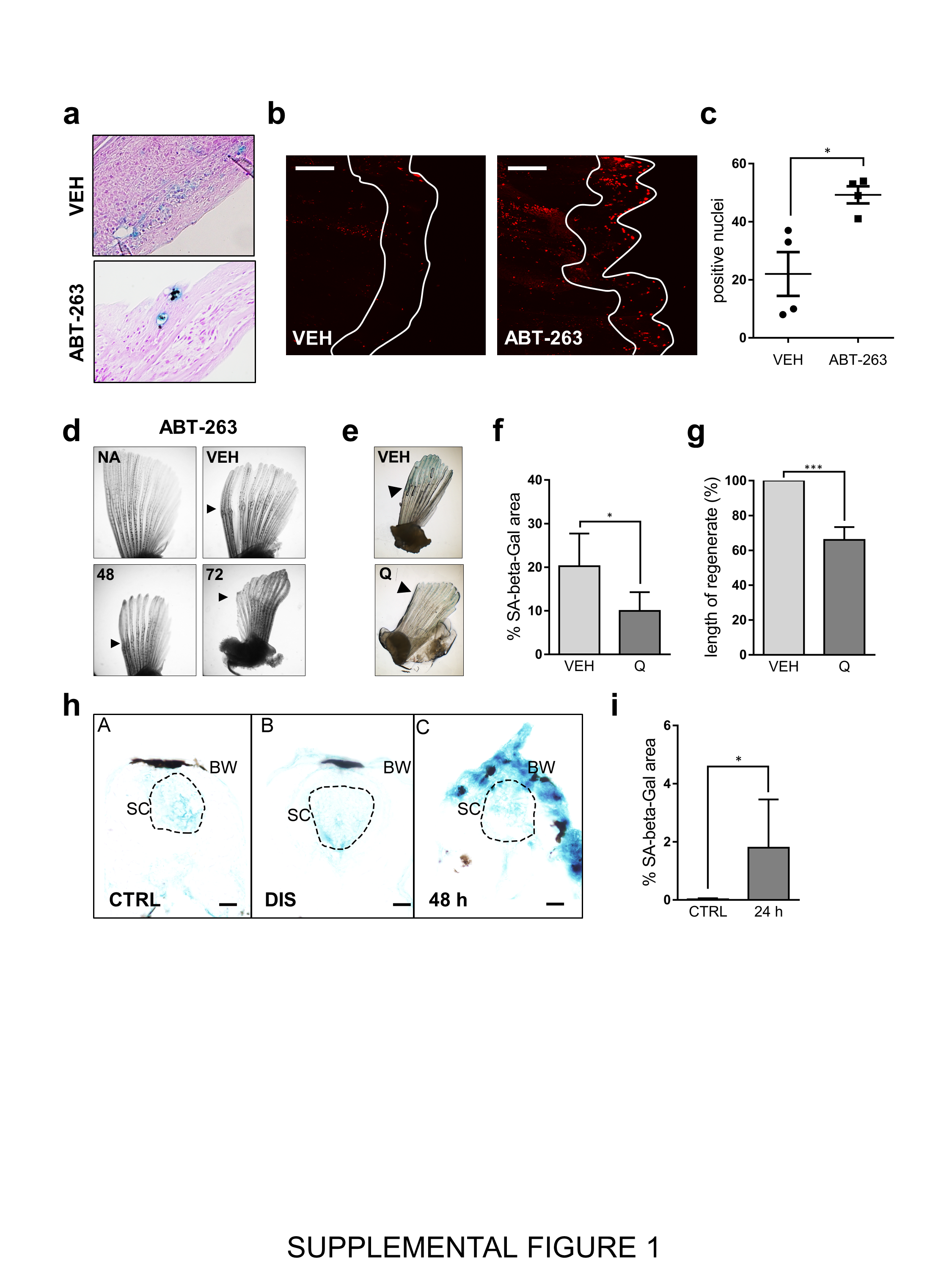


**Supplementary figure 1**. (a) Representative photomicrographs of sections of amputated fins (8 days post-amputation, dpa) stained for SAbetaGal after ABT-263 or vehicle (VEH) treatment. (b) Representative photomicrographs of amputated fins (8 dpa) stained for TUNEL after ABT-263 or vehicle (VEH) treatment. Scale bar: 150 µm. (c) Quantification of TUNEL positive nuclei. (d) Representative photomicrographs of non-amputated fin (NA) or regenerated fins at 8 dpa and after treatment with ABT-263 for 48 or 72 hours, or with vehicle (VEH). Arrowheads show the amputation plane. (e) Representative pictures of fins stained for SAbetaGal after Quercetin (Q) or vehicle (VEH) treatment. Arrowheads show the amputation plane. (f). Quantification of the SAbetaGal positive area in 8 dpa fins treated with Quercetin (Q) or vehicle (VEH). (g) Length of regenerate (%) reached by amputated fins at 8 dpa and after treatment with Quercetin (Q) relative to vehicle (VEH) treated fish. (h) Representative photomicrographs of transverse sections of 5 dpf zebrafish stained for SAbetaGal after spinal cord section. Control un-lesioned animal (panel A, CTRL); distal area (panel B, DIS) and injured area (panel C, 48h) of spinal cord sectioned animals. Abbreviations: BW: body wall, SC: spinal cord. Scale bars: 20 µm. (i) Quantification of the SAbetaGal positive area in regenerated caudal fins of larvae 24h after amputation, and in unamputated fins.
